# SDHB and CDK1 protein expression in metastatic pheochromocytoma and paraganglioma

**DOI:** 10.1530/EO-26-0035

**Published:** 2026-08-24

**Authors:** Lisa Gunnesson, Sinan Karakaya, Sofie Mohlin, Andreas Muth, Erik Elias

**Affiliations:** ^1^ Department of Surgery, Sahlgrenska University Hospital, Gothenburg, Region Västra Götaland, Sweden; ^2^ Department of Surgery, Institute of Clinical Sciences, Sahlgrenska Academy, University of Gothenburg, Gothenburg, Sweden; ^3^ Division of Pediatrics, Department of Clinical Sciences, Lund University, Lund, Sweden; ^4^ Lund Stem Cell Center, Lund University, Lund, Sweden; ^5^ Lund University Cancer Center, Lund University, Lund, Sweden; ^6^ Sahlgrenska Centre for Cancer Research (SCCR), Sahlgrenska Academy, University of Gothenburg, Gothenburg, Sweden

**Keywords:** PPGL, neuroendocrine tumors, metastasis, biomarkers

## Abstract

**Background:**

Pheochromocytomas and paragangliomas (PPGLs) are rare and mostly non-metastatic tumors originating from adrenal medulla and paraganglia. Metastatic PGLs have a worse prognosis, but currently, there are no established criteria to determine PPGL metastatic potential.

**Aim:**

The aim of this study was to investigate whether SDHB and CDK1 expression is associated with metastatic capacity in PPGL.

**Material and methods:**

A tissue microarray (TMA) was constructed from 175 tumors from a total of 149 unique PPGL patients treated at Sahlgrenska University Hospital. SDHB, CDK1, and proliferation index Ki-67 were assessed and correlated to metastatic capacity defined as a confirmed metastatic event.

**Results:**

Negative SDHB expression was more common in patients with metastatic PPGL and displayed a trend toward lower overall survival and correlation with higher Ki-67%. High CDK1 expression was not associated with any of the parameters mentioned above.

**Conclusion:**

Negative SDHB expression but not high CDK1 expression is associated with metastatic capacity in PPGL.

## Introduction

Pheochromocytomas and paragangliomas (PPGLs) are rare tumors arising from Schwann cell precursors in the adrenal medulla and extra-adrenal paraganglionic tissue, respectively (1). Although mostly non-metastatic, around 10–20% of patients develop recurrent or metastatic disease (2). In contrast to most neoplasms, they do not have certain pathological features of malignancy, thus making it difficult to determine which tumors will later metastasize, and even apparently sporadic cases have been observed with recurrence after many years (3). The treatment for all non-metastasized PPGLs is surgical resection (4), but better tools are needed for tailoring post-operative follow-up and prognosticating the individual risk of recurrent or metastatic disease. Several scoring systems have been developed aiming to improve distinction between metastatic and non-metastatic PPGLs, and both the PASS (5) and the GAPP score (6) are commonly used, but are, to some extent, user dependent and not entirely reliable (7, 8). Recently, loss of SDHB expression has emerged as a potential predictor of metastatic capacity and weak or complete loss of protein expression has been linked to poorer prognosis (9, 10, 11). Pierre *et al.* introduced COPPS (12) combining pathological tumor features (tumor size, necrosis, and vascular invasion) with immunohistochemistry of PS100 and SDHB, showing promising results, but further validation is needed. Additionally, in a recent ambitious effort to further understand what differentiates metastatic PPGL from non-metastatic PPGL, the expression of cyclin-dependent kinase 1 (CDK1) was proposed as a novel prognostic marker in PPGL (13). To our knowledge, CDK1 protein expression has not been evaluated in other patient cohorts. In this paper, we utilize a PPGL tissue microarray (PPGL-TMA) to assess the expression of CDK1 and SDHB in tumors from a clinical PPGL cohort and link protein expression levels to metastatic capacity.

## Materials and methods

### Patient cohort and TMA

The patient cohort and TMA have been described previously (14). In brief, the cohort comprised 149 patients diagnosed with PPGL at Sahlgrenska University Hospital, Gothenburg, Sweden, between 1984 and 2015, with corresponding tumor tissue samples. Clinical data were retrieved from patient charts, a pre-existing clinical database, and pathology reports. For TMA construction, representative areas from formalin-fixed, paraffin-embedded tumor blocks were selected by a board-certified pathologist, and tissue cores were transferred to recipient blocks. In total, 175 donor cores representing 149 primary tumors were included. Core quality was evaluated by eosin staining.

### IHC staining and scoring

#### CDK1

For CDK1 staining, FFPE tissue sections were deparaffinized, rehydrated, and subjected to heat-induced antigen retrieval in Tris-based antigen unmasking solution. Endogenous peroxidase activity was blocked with 3% hydrogen peroxide, followed by blocking of non-specific binding. Sections were incubated with mouse anti-CDK1 antibody (1:200; BD Biosciences, USA, Cat# 610038, RRID: AB_397454), followed by HRP-conjugated secondary antibody and DAB visualization. Slides were counterstained with hematoxylin, dehydrated, mounted, and scanned at 20× magnification using an Olympus VS120 slide scanner.

CDK1 staining was analyzed in QuPath (v0.3.0). TMA cores were identified using the dearrayer function and manually adjusted when needed. Nuclear staining intensity in tumor cells was scored as negative (0), weak (1+), intermediate (2+), or strong (3+). Cell detection and intensity thresholds were applied in QuPath and manually verified. Each slide was analyzed independently three times, and the final score was assigned after comparison of the three assessments.

#### SDHB and Ki-67%

For SDHB and Ki-67, 3–4 μm TMA sections were stained using Dako PT-Link and EnVision FLEX on a Dako Autostainer Link according to the manufacturer’s instructions. Antibodies used were SDHB (Invitrogen, USA, 459230, 1:100) and Ki-67 (Agilent, USA, IR62661, ready-to-use with a linker). Slides were scanned at 20× magnification using an Axio Scan Z.1. SDHB staining had been evaluated previously in connection with an earlier study (14). For the present study, SDHB negativity was reassessed by a blinded observer (E E), with complete agreement for negative cases. SDHB scoring data were not included in any analyses in the previous article.

### Statistics

The statistic tests used are detailed in corresponding results. In brief, Fisher’s exact test, the Man–Whitney U test, and log-rank test were used. Statistical analyses were performed using GraphPad Prism, version 10.2.0, GraphPad Software, USA, www.graphpad.com.

### Ethics

This study was approved by the Local Ethical Board of Gothenburg and performed in accordance with the guidelines and regulations of the Local Ethical Board of Gothenburg (ethical permit no. 833-18). Some patients included in the TMA had also been included for prospective tissue sampling, and in accordance with the ethics permit, we obtained informed consent for these patients. Also, in accordance with the ethics permit, patients where only archival material and clinical data were used were not required to provide informed consent.

## Results

### Characteristics of patients with PPGL with metastatic capacity

**Figure 1:**
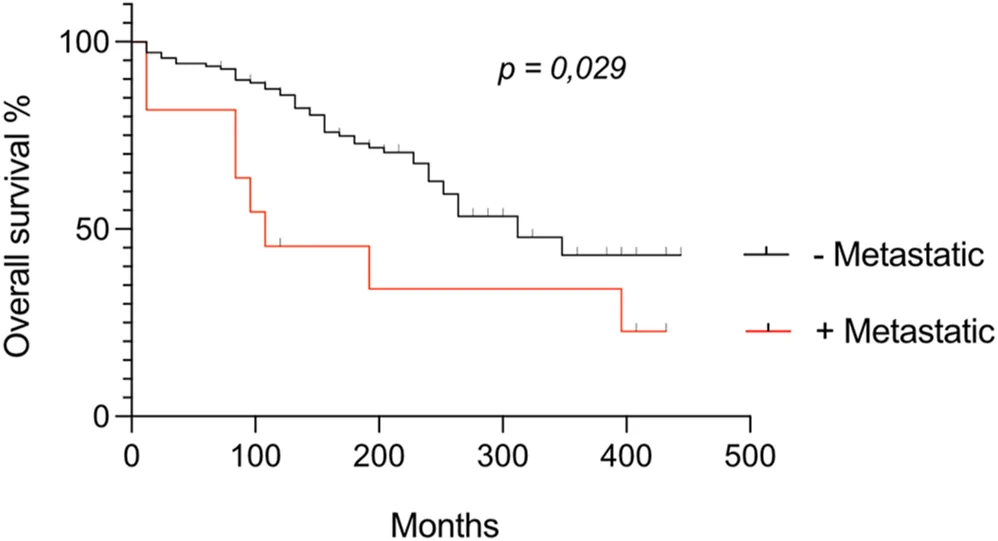
Overall survival in patients with metastatic disease (*n* = 11) compared with non-metastatic patients (*n* = 138) on the TMA.

Metastatic capacity was defined as patients with a confirmed metastatic event. This could represent a synchronous tumor (8/11) at the time of diagnosis or a metachronous tumor (3/11) detected at follow-up. Details regarding metastatic disease, time to recurrence, cause of death, and the treatment received are presented in Table 1. Overall survival in patients with tumors with metastatic capacity was significantly lower compared with other patients present on the TMA (*P* = 0.029, *n* = 11 vs 138) (Fig. 1).

**Table 1:** Clinical details for patients with metastatic PPGL in the Sahlgrenska TMA cohort (*n* = 11/149).

Age	Sex	Tumor type	Germline mutation	Presentation of metastatic disease	Recurrence-free survival	Metastasis site	Treatment	Survival (years)	Cause of death
52	M	PC	No	Metachronous	4	Bone	Surgery, chemotherapy	7	PPGL
40	M	PC	No	Metachronous	15	Local recurrence/lymph node	Surgery	54	Age
59	M	PGL	No	Synchronous	n/a	Bone	Surgery, chemotherapy, radiation	1	Suicide
61	M	PC	No	Synchronous	n/a	Bone	Surgery, MIBG, chemotherapy	1	PPGL
31	F	PC	No	Synchronous	n/a	Bone and lungs	Surgery, TKI, chemotherapy, radiation	3	PPGL
54	F	PGL	Unknown	Synchronous	n/a	Brain, lungs, bone, ovaries	Surgery, chemotherapy, radiation	8	PPGL
50	M	PGL	SDHB	Synchronous	n/a	Bone	Chemotherapy, radiation	7	PPGL
57	F	PGL	SDHB	Synchronous	n/a	Liver, bone	MIBG, PRRT (Lu), chemotherapy, radiation	1	PPGL
37	F	PC	No	Synchronous	n/a	Lymph node	Surgery	39	Unknown
32	M	PGL	No	Synchronous	n/a	Liver	Unknown	Unknown	Unknown
60	M	PGL	No	Metachronous	6	Unknown	Unknown	9	PPGL

### SDHB and CDK1 expression in PPGL

**Figure 2:**
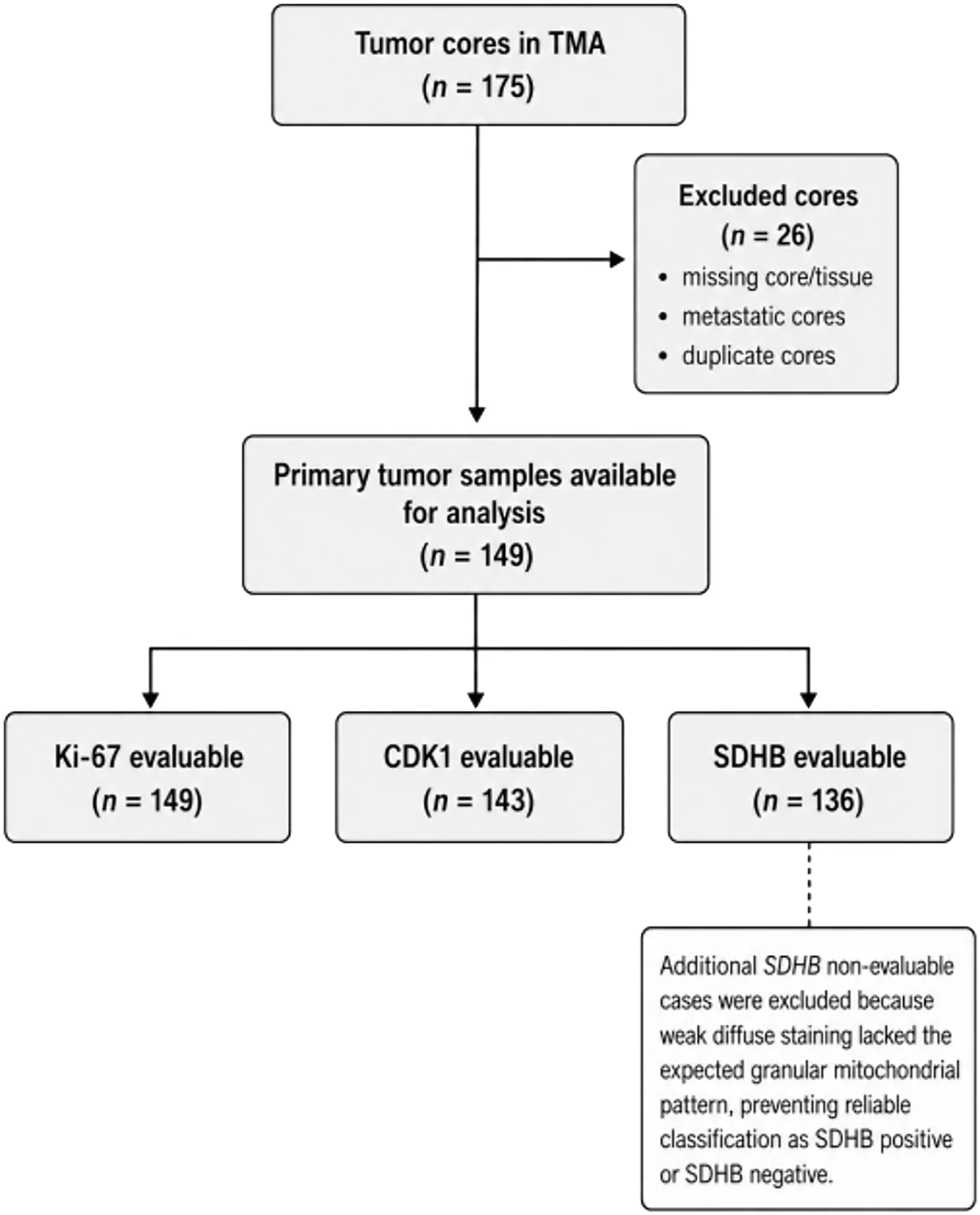
Flow chart of samples present for analysis.

A flow chart regarding samples available for further analysis is given in Fig. 2. Of the 175 tumor cores, 26 were excluded from the analysis because of missing tissue/core, metastatic tumor cores, or duplicate cores. This resulted in 149 primary tumor samples available for analysis. Ki-67 was evaluable in all 149 samples. Due to some missing cores after IHC staining, CDK1 staining was evaluable in 143 samples (6 missing cores after staining), whereas SDHB staining was evaluable in 136 samples. The lower number of evaluable SDHB cases was due to cases showing weak diffuse cytoplasmic staining without the expected granular mitochondrial staining pattern. Since retained SDHB expression is defined by distinct granular cytoplasmic/mitochondrial staining, these diffusely stained cases could not be reliably classified as either SDHB-retained or SDHB-loss and were therefore excluded from SDHB-specific analyses.

Patient characteristics of the TMA cohort have been published previously (14), and key characteristics are briefly summarized in Table 2.

**Table 2:** Distribution of samples according to age, location, and protein expression.

Patient cohort	Number of patients (%)	Mean age (years) at diagnosis (range)	Metastatic (%) Yes/no
PPGL	149 (100)	53 (15–83)	7/93
sPGL	13 (9)	54 (32–72)	46/54
HNPGL	13 (9)	47 (22–76)	0/100
PCC	123 (82)	51 (15–83)	4/96

**SDHB**	**SDHB+/SDHB−**	**Mean age (years) at SDHB+ vs SDHB−**	**Gender SDHB− Male vs female**

PPGL	125/11	52 vs 46	7 vs 4
sPGL	12/1		
HNPGL	13/0		
PCC	100/10		

**CDK1**	**CDK1 low/CDK1 high**	**Mean age (years) CDK1 low vs CDK1 high**	**Gender CDK1 high Male vs female**

PPGL	58/85	51 vs 51	42 vs 43
sPGL	4/9		
HNPGL	1/10		
PCC	53/66		

**Figure 3:**
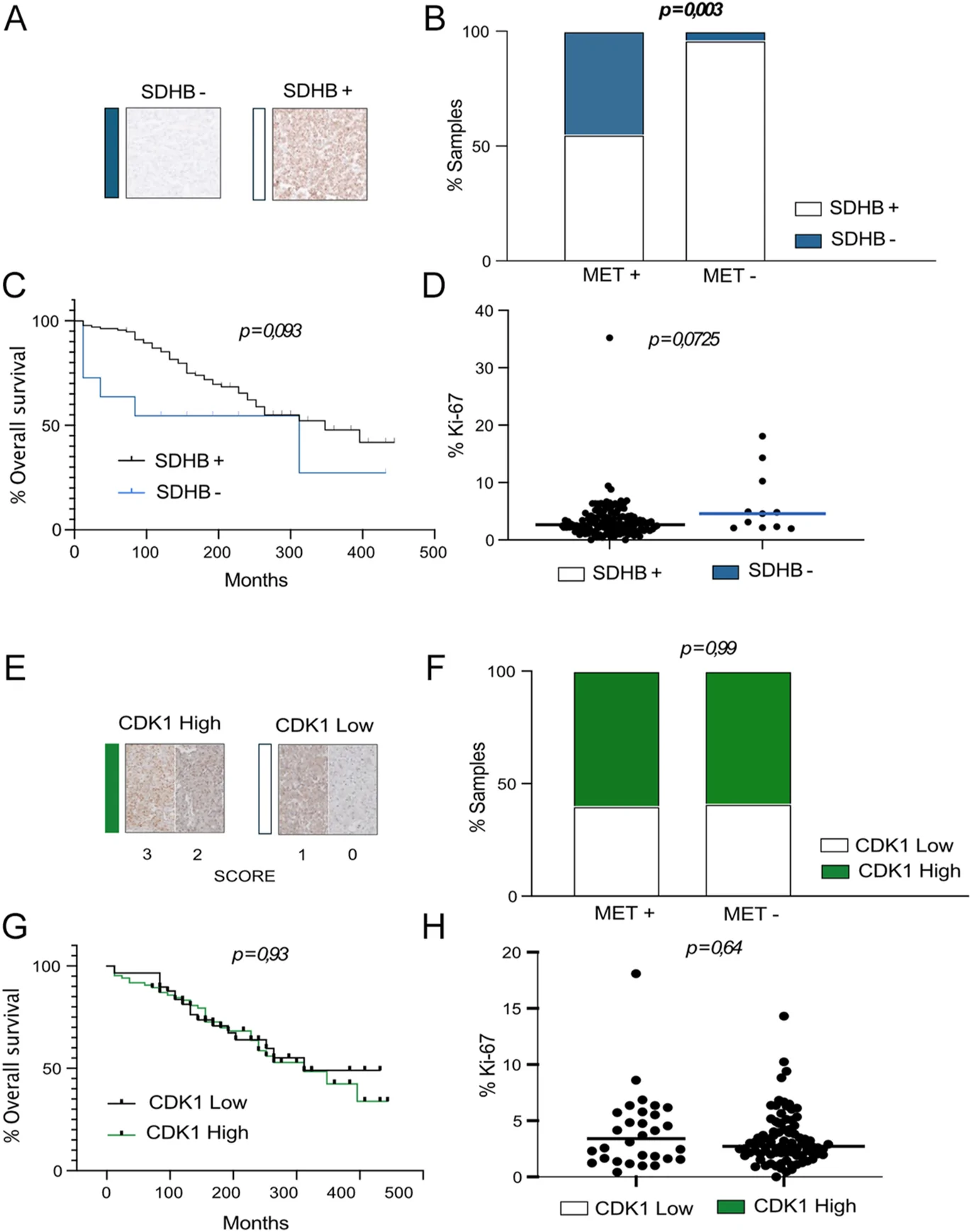
(A) Representative images of SDHB-negative (SDHB Neg) and SDHB-positive staining (SDHB Pos). (B) Fraction (%) of SDHB Neg and SDHB Pos tumors in patients with a confirmed metastatic event (MET+) or no metastatic event (MET−). (C) Overall survival (months) in relation to SDHB expression. (D) Ki-67% expression in tumors with SDHB-negative (SDHB Neg) and SDHB-positive staining (SDHB Pos). (E) Representative images of CDK1 high and CDK1 low staining and scores 0–3. (F) Fraction (%) of CDK1 high and CDK1 low tumors in patients with a confirmed metastatic event (MET+) or no metastatic event (MET−). (G) Overall survival (months) in relation to CDK1 expression. (H) Ki-67% expression in tumors with CDK1 high and CDK1 low.

Eleven out of 136 patients with tissue samples available for analysis displayed negative SDHB staining, i.e. no staining present across the whole sample. Examples of negative and positive staining are depicted in Fig. 3A. Zero out of 13 HNPGL, 10/110 PCC, and 1/11 sPGL samples were negative for SDHB staining (Table 1). Eighty-five out of 143 patients with tissue samples available for analysis displayed high CDK1 staining. Examples of high and low staining are depicted in Fig. 3F. High CDK1 expression was detected in 10/11 HNPGL, 66/119 PCC, and 9/13 sPGL samples.

### SDHB expression, metastatic disease, overall survival, and proliferation index

Negative SDHB expression was more common in patients with a confirmed metastatic event, 5/11 (metastatic) vs 6/128 (non-metastatic), *P* = 0.0003, Fisher’s exact test, Fig. 3B. Patients with negative SDHB expression had lower overall survival compared with patients with SDHB expression in the first two decades after diagnosis. However, assessing differences in survival up to the endpoint of follow-up (up to 400 months), the difference was statistically non-significant, *P* = 0.093, log-rank test (Fig. 3C). Patients with SDHB-negative tumors displayed a trend toward higher Ki-67% than patients with SDHB-positive tumors (median: 4.57% vs 2.64%, *P* = 0.0725, Mann–Whitney U; Fig. 3E).

### CDK1 expression, metastatic disease, overall survival, and proliferation index

High CDK1 expression was not significantly different in patients with a confirmed metastatic event compared with patients without a metastatic event, 6/10 (metastatic) vs 79/133 (non-metastatic), *P* = 0.99, Fisher’s exact test (Fig. 3F). Patients with high CDK1 expression had similar overall survival compared with patients with low CDK1, *P* = 0.93, log-rank test (Fig. 3G). Samples with high CDK1 expression had similar Ki-67% compared with samples with low CDK1 expression (median: 3.4% vs 2.7%, *P* = 0.64, Mann–Whitney U; Fig. 3H).

## Discussion

In the present study, we link negative SDHB expression to metastatic capacity in PPGL. The presence of metastases *per se* is the strongest independent factor predicting poor prognosis in patients with PPGL, and this is reflected in our cohort as well (15). Other clinical manifestations often connected to shorter disease-free survival are extra-adrenal tumor location, voluminous tumors, and norepinephrine (NE) and/or dopamine secretion (16, 17). In our cohort, as previously described (14), we found clear overrepresentation of extra-adrenal tumors in the metastatic group (46 vs 4% of PCCs) and a slightly higher proportion of NE hormone profile. However, these factors are not sufficient to safely triage metastatic from non-metastatic tumors (4).

The role of genetics in PPGL development is well established, and no other human tumor shows a higher rate of germline mutations (18, 19). The involvement of SDHB mutations in PPGL tumorigenesis was first described in the early 21st century (20, 21) and has been shown to increase the risk of developing PPGL and metastatic disease with a poorer prognosis (22, 23). However, a germline mutation in the SDHB gene is not always the causative factor leading to loss of protein expression, and negative staining can reflect other mutations or epigenetic alterations (24, 25). Some studies find the sensitivity of IHC superior to that of mutational status in connecting SDHB to development of metastatic disease (11).

In contrast to negative SDHB staining, high or low CDK1 protein expression did not differentiate between metastatic and non-metastatic tumors and was not associated with worse prognosis or a more aggressive tumor phenotype in our patient cohort. In a recent publication, high CDK1 protein expression was linked to metastatic potential, but the association was considerably stronger at the transcriptome level. Additionally, CDK1 expression was recommended to be assessed in conjunction with total mutational burden, MSI score, and gain of chromosome 5 to provide prognostic information (13). Although CDK1 expression does not appear to be associated with metastatic capacity in our cohort, it could still provide valuable information in the above context. A recent study has shown a positive correlation between CDK1 gene expression and tumor size but it was not linked to metastatic potential (26). In comparison to negative SDHB staining, high CDK1 protein expression seems to provide significantly less prognostic information.

The strengths of this study include a relatively large TMA with material from 149 individual PPGL patients treated at our institute, including both primary tumors and metastases. However, only 11 patients were found to have SDHB-negative tumors, which increases the risk of type 2 errors in the statistical analysis, i.e. that true differences between groups fail to reach statistical significance due to a small sample size.

Also, the limited sample size and heterogeneous nature of PPGL patients with metastatic disease in our cohort limit the ability to assess negative SDHB expression as a potential prognostic biomarker. The trend toward lower overall survival in the SDHB-negative cohort is likely linked to metastatic disease being more common in this group, and it would require a much larger patient cohort to assess whether negative SDHB expression could represent an independent prognostic biomarker. Assessing the independent impact of negative SDHB expression would also require a multivariate analysis considering several additional aspects, such as treatment regime, tumor size, proliferation, and age, which is not feasible only utilizing our cohort. Ideally, a future collaborative multicenter study could gather sufficient sample size to explore these concepts further.

In summary, lack of SDHB expression but not high CDK1 was linked to metastatic capacity in the studied clinical PPGL cohort.

## Declaration of interest

The authors declare that there is no conflict of interest that could be perceived as prejudicing the impartiality of the work reported.

## Funding

This work was supported by The Mrs Berta von Kamprad’s Foundation, the Swedish Cancer Society, the Swedish Childhood Cancer Fund, the Lions Cancer Fund West, and institutional research funds from the Department of Surgery, Sahlgrenska University Hospital.

## Author contribution statement

SK, LG, EE, AM, and SM conceived the study; SK and LG curated the data and performed formal analysis and investigation; SK, LG, EE, and SM designed the methodology; SM, EE, and AM contributed resources; SK, LG, and EE performed validation; EE performed visualization; administered the project; and wrote the original draft of the manuscript; SK, LG, EE, AM, and SM reviewed and edited the manuscript; EE and SM supervised the study; and EE, AM, and SM acquired funding.

## Data availability

The data that support the findings of this study are not publicly available due to requirements of the approved ethics permit, but anonymized data are available from the corresponding author upon request.

## Statement of ethics

This study was approved by the Central Ethical Review Board in Gothenburg (Dnr. 833-18 and 2023-05593-02).
